# Clinical Aspects of Manic Episodes After SARS-CoV-2 Contagion or COVID-19

**DOI:** 10.3389/fpsyt.2022.926084

**Published:** 2022-06-15

**Authors:** Antonio Del Casale, Martina Nicole Modesti, Ludovica Rapisarda, Paolo Girardi, Renata Tambelli

**Affiliations:** ^1^Department of Dynamic and Clinical Psychology, and Health Studies, Faculty of Medicine and Psychology, Sapienza University, Rome, Italy; ^2^Unit of Psychiatry, ‘Sant'Andrea' University Hospital, Rome, Italy; ^3^Faculty of Medicine and Psychology, Sapienza University, Rome, Italy

**Keywords:** COVID-19, SARS-CoV-2, manic episode, bipolar disorder, neuroinflammation, neuroinvasion

## Abstract

As COVID-19 pandemic spread all over the world, it brought serious health consequences in every medical field, including mental health. Not only healthcare professionals were more prone to develop anxiety, depression, and stress, but the general population suffered as well. Some of those who had no prior history of a psychiatric disease developed peculiar symptoms following infection with SARS-CoV-2, mostly because of psychological and social issues triggered by the pandemic. People developed traumatic memories, and hypochondria, probably triggered by social isolation and stress. Infection with SARS-CoV-2 has influenced the mental health of psychiatric patients as well, exacerbating prior psychiatric conditions. In this review, we focus on analyzing those cases of mania in the context of bipolar disorder (BD) reported after COVID-19 disease, both in people with no prior psychiatric history and in psychiatric patients who suffered an exacerbation of the disease. Results have shown that COVID-19 may trigger a pre-existing BD or unmask an unknown BD, due to social and psychological influences (decreased social interaction, change in sleep patterns) and through biological pathways both (neuroinflammation and neuroinvasion through ACE-2 receptors expressed in the peripheral and central nervous systems (PNS and CNS respectively). No direct correlation was found between the severity of COVID-19 disease and manic symptoms. All cases presenting severe symptoms of both diseases needed specific medical treatment, meaning that they concur but are separate in the treatment strategy needed. This review highlights the importance of a now widespread viral disease as a potential agent unmasking and exacerbating bipolar mood disorder, and it can hopefully help physicians in establishing a rapid diagnosis and treatment, and pave the road for future research on neuroinflammation triggered by SARS-CoV-2.

## Introduction

On December 31st, 2019, several cases of atypical pneumonia arose in Wuhan, China, later found as being caused by a novel coronavirus called Severe Acute Respiratory Syndrome CoronaVirus-2 (SARS-CoV-2) (1). The World Health Organization classified the epidemic as a global pandemic on March 11th, 2020. As the pandemic was spreading, several important consequences on the mental health of the world population were emerging (2–4). Among these consequences, high rates of depression and anxiety among health professionals need to be highlighted (5), as well as anxiety, depression, and stress-related disorders in the general population (6, 7), psychological symptoms related to social isolation, especially in the elderly, poor, and subjects with difficulties in accessing or handling technology, such as telephone or Internet connections (7). Other symptoms that could arise in the general population are related to social isolation and quarantine (3, 8, 9), unemployment and financial difficulties (3, 9). Some of the psychological issues triggered by the pandemic are specific to infected patients and may include hypochondriac ideas, stigma-related concerns, amnesia, and traumatic memories of severe illness (4). Nevertheless, mental conditions have witnessed a change in paradigm, as the environment played a certain role in triggering some diseases, as previously stated, but the virus itself has the potential to influence the development of some neurological and psychiatric sequelae, as a direct effect of the coronavirus infection of the CNS and PNS, or an indirect effect of medical therapy or abnormal immune response, or a combination of these factors. Patients admitted to the hospital for severe SARS-CoV-2 infection can show different neurological symptoms, more commonly delirium. Different mental disorders may manifest in the subsequent months, including post-traumatic stress disorder, depression, anxiety, and fatigue. COVID-19 can be associated with delirium, agitation, and symptoms of depression, anxiety, and insomnia (4). The reason why this happens finds its roots in microbiological processes: the access of SARS-CoV-2 into human host cells is mediated by the angiotensin-converting enzyme II (ACE-2) receptor, mainly expressed in the lungs and gastrointestinal tract. This is also expressed in brain endothelial cells, which are a hypothetical route of entry into the CNS for coronaviruses (10, 11). Even if severe neurological and psychiatric direct consequences of SARS-CoV-2 appear to be rare, considering the prevalence of the pandemic, many people worldwide may have been affected (4, 12). Most of all, there is evidence of an overall increased vulnerability of patients with a primary diagnosis of bipolar disorder (BD) compared to the general population, despite some studies report a lower degree of distress in this population during the first month of the pandemic compared to a previous baseline (13, 14) Different COVID-19-related stressors can impact BD, mainly including social isolation, restrictive measures, lifestyle, biological circadian rhythms changes, and infection-related concerns (15). Furthermore, the access to mental health services among BD patients had become more difficult during some phases of the pandemic. Conversely, BD might indirectly worsen the risk of acquiring SARS-CoV-2 infection (15, 16). Neuroinvasion of the virus may represent a potential etiology for BD in absence of any other biological, psychological, and social precipitating factors. Nevertheless, some citokines appear to be involved in the development of psychiatric symptoms: it has been suggested that some may be specific for manic state (IL-2,4,6), and for depression (IL-6) (17, 18). Some authors have indeed suggested the particular need to develop standardized laboratory panels that include inflammatory markers (IL-6, TNF-α), cerebrospinal fluid (CSF) testing, and SARS-CoV-2 antibody assays to entirely understand the etiology of neuropsychiatric complications of SARS-CoV-2 infections and the pandemic itself (19). Neuroinflammation might be related to BD symptoms and is infrequently an etiological factor. An aberrant neuroglial function may be responsible for some neuronal miswiring that is consistent with psychotic symptoms frequently observed in BD (20). Manic episodes manifested during or after Covid-19 can arise both as an onset of BD and as a relapse. The main purpose of this article is to provide a review of the scientific literature focused on the clinical and biological correlates of cases of mania manifested in conjunction with or after SARS-CoV-2 infection.

## Methods

**Figure 1:**
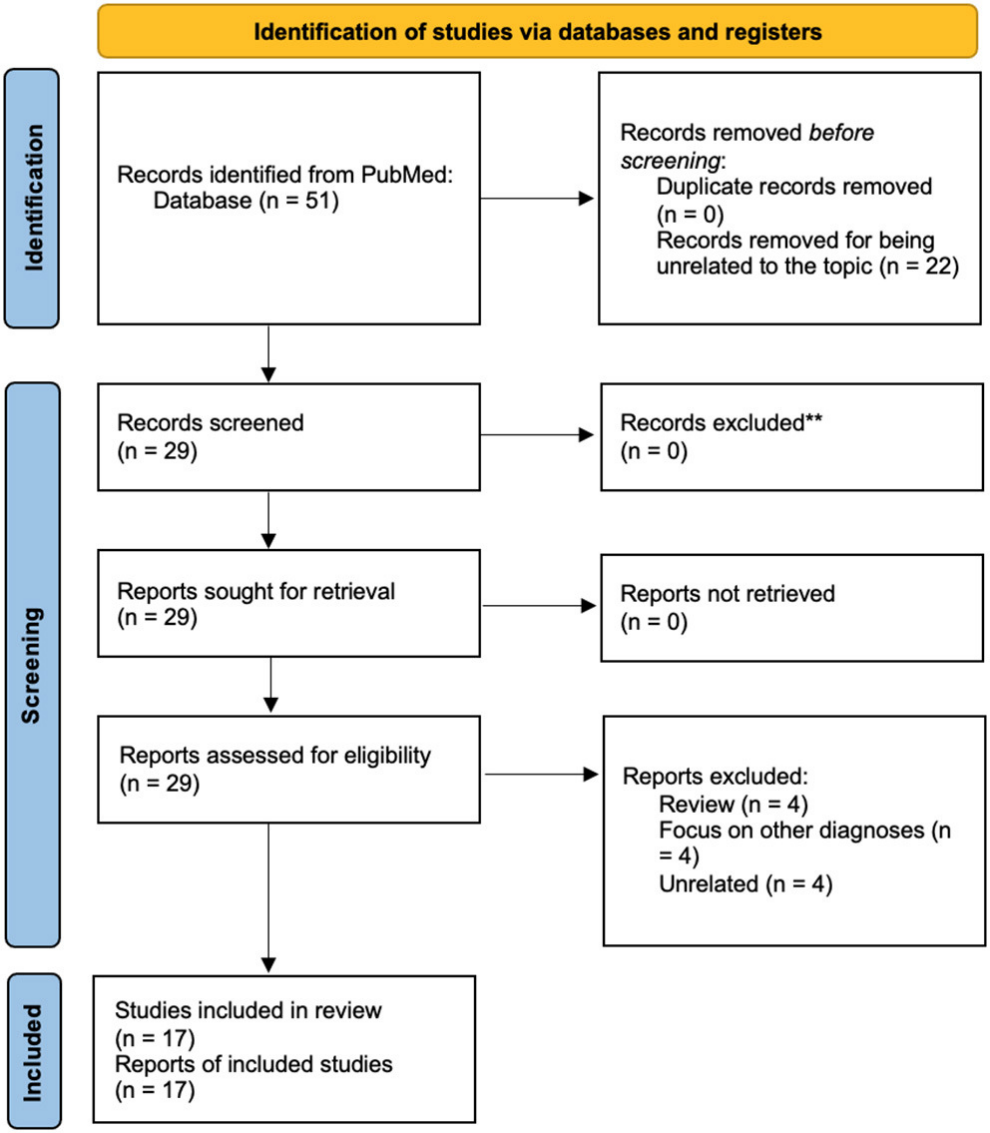
PRISMA 2020 flow diagram of the systematic review. For more information visit: http://www.prisma-statement.org/.

On April the 18th, 2022, we conducted a first research on PubMed with the title/abstract specification, using the terms “(mania OR manic) AND (COVID OR SARS-CoV-2)” in the search bar. For eligibility, we included randomized controlled studies, case-control studies, and case reports focused on the issue. We excluded reviews, other types of articles and other studies that did not focus on the main topic, such as BD following another medical condition. The system provided 51 articles, of which we excluded 22 for low relevance. Hence, we assessed 29 articles for eligibility, excluding 12 articles for not respecting the inclusion criteria (4 reviews, 4 unrelated to the topic, and 4 focused on other diagnoses). We finally included 17 articles in the qualitative analysis (see PRISMA flow diagram in Figure 1).

## Results

The main results are summarized as follows in Table 1 below.

**Table 1:** Studies of manic episodes comorbid with ymrsSARS-CoV-2.

**Article**	**Sample**	**Main SARS-CoV-2 related symptoms**	**Main manic symptoms**	**Therapy**	**Main clinical and pathophysiological correlates**
Iqbal et al. (21) Retrospective case series	15 cases of COVID-19-associated mania or hypomania Inclusion criteria: - Patients aged ≥ 18 years - Positive real-time polymerase chain reaction test for SARS-CoV-2 during hospitalization. Mean age: 40 years (age range: 23–66) Gender: 14 men 1 woman Psychiatric history: – 6 with bipolar disorder – 2 with psychosis – 1 with unipolar depression – 6 without a past psychiatric history Significant pandemic-related psychosocial stressors prior to admission: – Present: 9 cases – Absent: 6 cases Comorbidities: 1 patient had epilepsy, which was well controlled. 1 patient with BD had brain metastases	– Asymptomatic: 10 patients – Mild COVID-19: 2 – Mild COVID-19 pneumonia: 1 – Severe COVID-19 pneumonia: 2 – Raised peripheral inflammatory markers: 7 – Mild white matter ischaemic changes: 3	Insomnia (13 subjects), elation (10), behavioral disorder (10), delusion (9), irritability (9), agitation (8), aggressive behavior (8), anxiety (7), impaired concentration (5), persecutory beliefs (5), auditory hallucinations (5). Consultation-Liaison diagnoses: – Mania: 12 patients – Hypomania 3 patients	Steroids prescribed: – Yes 3 – No 12 Psychotropic medications prescribed: – Olanzapine: 12 – Benzodiazepines (lorazepam or clonazepam): 9 – Haloperidol: 6 – Antihistamines (diphenhydramine/promethazine): 5 – Valproate: 4 – Zolpidem: 3 – Quetiapine: 2 All patients responded well to standard treatment for mania or hypomania. Upon discharge from COVID19-designated hospitals: – 5 patients were transferred to a psychiatry hospital for the treatment of ongoing manic symptoms – 10 patients were discharged to their homes.	Potential mechanisms by which SARS-CoV-2 could be a risk factor for mania or hypomania: – Psychosocial stress related to the pandemic, social isolation, and financial difficulties (9 cases). – Raised peripheral inflammatory markers (7 cases) – A history of bipolar affective disorder (6 cases, of which there was evidence of poor medication adherence in 3 cases). – Steroids were prescribed to 3 patients. Steroids can cause mania and have been implicated in some reports of COVID-19-associated mania – Hypoxia, inflammation, and a hypercoagulable state may be risk factors. – Neuroinflammation may be the most plausible correlate of manic states related to SARS-CoV-2.
Haddad et al. (22) Case report	A 30-year-old woman with no family history of mental health problems. No current or past pathologies.	A mild cough and diffuse bodily aches RT-PCR: positive Laboratory tests: elevated CRP, ferritin, LDH	The patient showed features of both delirium and mania. Manic features: elevated mood, decreased need for sleep, grandiose ideation, increased talkativeness, pressured speech, flight of ideas and distractibility. Classic features of delirium: acute disturbance of attention, awareness and cognition, misidentification of family members, brief visual hallucinations, reduced attention, disorientation to time and behaviors that implied reduced awareness, including undressing inappropriately and ingesting body wash (she denied an attempt to harm herself) → diagnosis of delirious mania	Dexamethasone, Ceftriaxone, Enoxaparin, Remdesivir, oxygen therapy Lorazepam 2 mg per day, Quetiapine 300 mg per day → at discharge: Quetiapine 500 mg per day → Reduction to quetiapine 200 mg at night → stopped 4 months after discharge (effective treatment).	Although the patient showed some symptoms consistent with hyperactive delirium, the manic symptoms were not explained by delirium. Psychiatric symptoms started soon after she developed physical symptoms of COVID-19 and received a positive PCR test. The duration of symptoms (9 days in total) was consistent with COVID-19. The raised inflammatory markers provide a plausible aetiological mechanism by which COVID-19 could cause neuropsychiatric symptoms. The association of delirious mania with COVID-19 was coincidental with the former representing the first episode of BD.
Jiménez-Fernàndez et al. (23) Case report	A 71-year-old retired male patient with no medical history of major affective disorders	Fever, mild cough, dizziness CT: cortico-subcortical retraction pattern Blood tests: slight unspecific abnormalities PCR SARS-CoV-2 test: positive	Admitted to ED for confusion, elevated mood, logorrhoea, excessive motor activity, global insomnia, megalomaniacal delusions, sexual disinhibition, prodigality, and cognitive symptoms.	Corticosteroids 1 month back for a recurrent varicella-zoster lesion Quetiapine 75 mg per day, clonazepam 1 mg per day.	Possible conditioning factors: -infection with COVID-19 (neuroimmune response, biochemical alterations, neuroinvasion) -treatment with corticosteroids.
Kozian and Chaaban (24) Case report	85-year-old patient with no psychiatric history	COVID-19, mild symptoms	Elevated mood, increased drive, and behavior		
Kummerlowe et al. (25) Case report	A 56-year-old Caucasian male with no past personal or family psychiatric history	Mild neutrophilia, thrombocytosis, and elevated ESR and CRP SARS-CoV-2 nucleocapsid total antibody was positive (not vaccinated)	Presented to ED for new-onset odd and erratic behavior preceded by a 4-week period of decreased need for sleep, fluctuating mood, increased energy, distractibility, overvalued religious ideation that he was a prophet, conceptual disorganization, auditory hallucinations but demonstrated insight. Received a diagnosis of brief psychotic disorder → rapid improvement → discharged after 24 hours → 10 days after re-presented to the ED with labile mood, increased energy, pressured speech, talkativeness, distractibility, overvalued religious thought, and brain fog → manic episode	Not treated with corticosteroids or antibiotics Risperidone 1 mg and Lorazepam 1 mg daily: rapid response (24 h) 2° access to ED: restarted Risperidone 1 mg increased up to 2 mg daily.	Symptoms rapidly improved following treatment with atypical antipsychotics, but maintenance treatment needs to be considered even after the early remission as with typical manic episodes. Not certain whether this case may be correlated with SARS-Coronavirus-2 itself or immune response. COVID-19 infection could trigger an initial manic episode; SARS-CoV-2 could penetrate the blood-brain barrier and stimulate the production of cytokines (TNF-α, IL 1 and 6, and INF-α).
Lu et al. (26) Case report	A 51-year-old male patient without a past or family history of mental disorders	Laboratory tests: leukopenia, increased plasma levels of IL-6, IL-10, and CRP in the acute phase of the illness. Positive for SARS-CoV-2 IgG antibody in CSF. Brain MRI: small ischemic lesions located at the basal ganglia and semioval center, suggesting no major pathological changes in the brain.	On illness day 17 he showed excitement, logorrhoea, irritability, ideas of grandiosity, and decreased need for sleep. YMRS= 36	Arbidol, Moxifloxacin, Darunavir and Cobicistat Tablets, and Methylprednisolone Haloperidol, Olanzapine.	Possible risk factors for mania: – neuroinvasive potential of SARS-CoV-2 inducing CNS symptoms. – SARS-CoV-2 IgG in CSF as possible evidence of a past CNS infection – inflammation (increased IL-6, IL-10, and CRP in the acute phase of the illness) – Moxifloxacin – Methylprednisolone.
Mahapatra and Sharma (27) Case series	A 48-year-old married man, with secondary level education, of middle socioeconomic status, with well-adjusted premorbid functioning. No history of mental disorders. Father was possibly affected by BD.	COVID-19 requiring hospitalization; meanwhile, his mother died due to COVID-19.	After 2 weeks he showed decreased need for sleep, logorrhoea, irritable mood, and ideas of grandiosity. YMRS=27	Olanzapine 15 mg per day; Clonazepam 1 mg per day optimized over 1 week → clinical response over the next 2 weeks.	Possible risk factors for mania: – bereavement (and impossibility of attending a funeral) – genetic vulnerability – COVID-19.
Mawhinney et al. (28) Case report	A 41-year-old man, with no significant medical history, reported a previous cannabis-induced severe transient mood reaction (no further use since then). Sister affected by BP.	Presented to ED with severe headache and a 10-day history of dry cough and fever. He was positive for SARS-CoV-2, chest X-ray showed pneumonitis. CRP and neutrophils were initially raised. Normal neuroimaging and lumbar puncture.	He received a diagnosis of a manic episode, showing decreased sleep, agitation, flight of ideas, hypochondriasis, sexual disinhibition, elevated mood, pressured speech, and persecutory, mystical, and grandiose ideas, needing heavy sedation and intensive care.	Antimicrobial and antiviral treatment for 48 hours; he was extubated after less than 24 hours. Clinical response of manic symptoms to olanzapine, antipsychotics and benzodiazepines.	Possible risk factors for mania: – Neuroinvasion of the virus The development of validated assays for SARS-CoV-2 in the CSF may help to determine the neuroinvasive potential of the virus.
Panda et al. (29) Case series Case report 1	A 58-year-old man affected by severe BD, diabetes.	COVID-19 pneumonia with fever, cough, and breathlessness Inflammatory markers were raised	Excessively cheerful, logorrhoea, disinhibited behavior, reduced sleep, grandiose ideas. Diagnosed as BD, current manic episode	Amoxicillin, Dexamethasone, Remdesivir Last 3 months before COVID-19: Sodium valproate 1500 mg per day, chlorpromazine 100 mg per day. After infection, chlorpromazine was stopped, and the patient was treated with haloperidol 10 mg per day, Sodium Valproate 1500 mg per day.	Possible risk factors for mania: – production of a high amount of pro-inflammatory factors. This proinflammatory state leads to relapse in patients with BD – iatrogenic factors, including corticosteroids and antibiotics – stress due to diagnosis of COVID-19, isolation, and hospitalization.
Reinfeld and Yacoub (30) Case report	A 50-year-old, married, employed man, no psychiatric history	Low fever, tachycardia, elevated blood pressure Chest CT: bilateral pneumonia Laboratory test: elevated ESR, CRP, ferritin, D-Dimer PCR SARS-CoV-2 test: negative, but 2 weeks after discharge COVID-19 IgG were positive	Delirium, mania and catatonia. Admitted to ED reporting aggressiveness, episode of staring, decrease of sleep, decreases speech, beliefs that he was responsible for pandemic, and suicidal ideation. During hospitalization continued to pace, paranoid delusion, rapid and pressured speech, incoherent behaviors, staring episode, intermittently mute, hyperactivity, fluctuating orientation.	Broad-spectrum antibiotics Lorazepam → no response Elettroconvulsive Therapy: bifrontal treatments over 2 weeks, 0.5 ms-70 Hz (anesthetic and neuromuscular blocker used: succinylcholine and methohexital, later replaced by etomidate) → symptoms gradually improved, after 6th treatment euthymic, normal sleep-wake cycle, no excitement, normal speech, no delusion, no suicidal ideation.	Possible risk factors for mania: – Neuroinflammation (elevated acute-phase proteins and cytokines, in particular IL-6, TNF-alpha) – Not completely understood connection with SARS-CoV-2 infection.
Russo et al. (31) Case report	A 60-year-old woman with a diagnosis of major depression. Family history was positive for cognitive deficits (maternal grandmother), delusions (mother), and major depression (two siblings).	Swab positive, asymptomatic for covid-19. Negative neurological exam Negative MRI Normal EEG Laboratory tests: mild anemia	Delusions consisting of mold growing everywhere (threw away several pieces of furniture and bought large amounts of cleaning products to deep clean her house), hallucinations (her dead mother ordered her to clean the tombstones of all her relatives to be safe from COVID-19), aggressive behavior, restless, and insomnia. Brought to a psychiatric ward, at admission she showed euphoria, accelerated speech, racing thoughts, logorrhoea, and distractibility. Diagnosed with mania with psychotic features triggered by SARS-CoV-2 infection.	Prednisone 1 mg/kg for two days. Haloperidol 8 mg per day → after 2 weeks no notable changes → switched to Clotiapine 8 months later she can recall hallucinations and delusions. Increased activity, buying sprees, engagement in goal-directed pursuits have disappeared.	Possible risk factors for mania: – SARS-CoV-2 infection – a steroid-dependent mechanism was ruled out.
Sen et al. (32) Case report	A 33-year-old high-school-graduated female patient with no previous neurological or psychiatric history and no prior alcohol or substance abuse	Sore throat and fever (37.8 C) SARS-CoV-2 IgM antibodies CT: bilateral ground-glass opacities Blood screening: increased levels of white blood cell count, C-reactive protein, fibrinogen, ferritin, and D-Dimer MRI: hyperintense signal in the splenium of the corpus callosum with decreased apparent diffusion coefficient: possibly presence of cytotoxic oedema	Admitted to ED reporting an acute onset of insomnia, irritability, and paranoid delusions; logorrhoea and increased psychomotor activity; anxiety and dysphoric mood. Hospitalized with a diagnosis of an acute manic episode. YMRS = 43	COVID-19: Hydroxychloroquine 400 mg per day; Favipiravir 1200 mg per day Haloperidol 20 mg per day; Biperiden 10 mg per day → during hospitalization switched to a daily dose of 20 mg of Olanzapine.	Structural changes of the splenium could be associated with insomnia, irritability, behavioral changes, and psychosis. No corticosteroid was administered, which supports the hypothesis that the manic symptoms may be related to the infection itself. Possible risk factors for mania: – COVID-19 related neuroinflammation and release of pro-inflammatory cytokines in the CNS (TNF-a, IL-1 and IL-6).
Uvais and Mitra (33) Case report	A 22-year-old unmarried woman, no family psychiatric history, no medical comorbidity, no significant life stressors, personal history of obsessive-compulsive symptoms	COVID-19	Admitted to a psychiatric department for a diagnosis of obsessive-compulsive disorder comorbid with moderate depressive episode treated with fluoxetine. After 2 days from COVID-19, she showed talkativeness, overactivity, sexual disinhibition, reduced need for sleep, gender incongruence, and irritability, for which she received a diagnosis of manic episode.	Sodium valproate gradually increased to 1000 mg per day; olanzapine gradually increased to 10 mg per day. Improvement in a month.	It is uncertain if the COVID-19 influenced the illness course. Gender incongruence for the past 5 years may have been covered up for social stigma.
Uvais and Moiden (34) Case report	A 36-year-old male with type 2 diabetes, no personal or family psychiatric history, history of substance abuse	Fever, cough, and diarrhea.	Disorientation, irritability, delusion with religious contents, grandiosity, urinary incontinence, impaired appetite, decreased need for sleep, increased energy and motor activity. Diagnosed with Catatonic disorder due to a general medical condition (delirious mania associated with COVID-19 infection).	Antibiotics Olanzapine 2,5 mg/day → remained disoriented, manic symptoms exacerbated Olanzapine increased up to 5 mg → symptoms resolved in about a week.	Delirium and manic symptoms developed in middle age instead of young adulthood. Possible mechanisms could be: – SARS-CoV-2 neuroinvasion – Immunological response and the related effect on the CNS – Hyper-inflammatory state (ferritin, CRP, IL-6).
Uvais (35) Case report	A 45-year-old woman with a severe depressive episode. Family history of BD (maternal aunt).	COVID-19 pneumonia	Logorrhoea, irritability, increased energy level, reduced need for sleep; diagnosed with a current manic episode with psychotic symptoms.	Bilevel positive airway pressure, oxygen therapy and oral steroids (quetiapine was stopped) Injection of low molecular weight heparin for 5 days for pulmonary embolism Risperidone 2 mg per day; lorazepam 3 mg per day.	Possible risk factors for mania: – Steroid-induced mechanism – COVID-19 related stress (hospitalization, isolation) – Neurotropism for the virus – Immunologic response associated with COVID-19 CNS infection.
Uzun et al. (36) Case report	A 16-year-old boy with cerebral palsy and no previous psychiatric disorder nor family history	mild COVID-19 symptoms	10 days after recovery he presented excessive speaking, euphoria, irritability, increased energy and decreased sleep and appetite.	Risperidone and after 1 month Lithium was added → symptoms disappeared → Maintenance therapy: Risperidone 3 mg/day and Lithium 1200 mg/day → manic symptoms did not recurred over 4 months.	Possible risk factors for mania: – COVID-19 related neuroinflammation and release of pro-inflammatory cytokines in the CNS – psychosocial stress due to COVID-19 – neurological disability.
Varsak et al. (37) Case report	A 64-year-old woman with no psychiatric history	Fever, myalgia, headache, diarrhea, taste and smell alterations. CT: bilateral patchy shadows and ground-glass opacity.	On day 3 of hospitalization: cheerful and irritable mood, logorrhoea, aloud singing, throwing things out of the window, and grandiose and mystic delusions. On day 7: agitation, hostile behaviors, and aggressiveness. YMRS= 43.	Hydroxychloroquine, Enoxaparin sodium, Salbutamol, Methylprednisolone Haloperidol 20 mg and Biperiden 5 mg i.m. for agitation → Zuclopenthixol acetate, Haloperidol 10 mg i.m. → Zuclopenthixol decanoate/2 weeks, Olanzapine 20 mg per day, Biperiden 4 mg per day → Olanzapine 10 mg per day.	The patient experienced first-episode mania during the COVID-19 treatment. The absence of psychiatric history and the first manic episode during the treatment of COVID-19 led to associating this case to the SARS-CoV-2 infection.

*BD, bipolar disorder; CNS, central nervous system; COVID-19, coronavirus disease 2019; CRP, c-reactive protein; CSF, Cerebrospinal fluid; CT, computed tomography; ED, emergency department; ESR, erythrocyte sedimentation rate; MRI, magnetic resonance imaging; PCR, polymerase chain reaction; SARS-CoV-2, severe acute respiratory syndrome coronavirus*.

All studies are observational, being case-reports/case-series. Only traditional medical treatments were employed for the diseases. All studies regarded mania manifestations in different scenarios, and patients from cases analyzed shared a diagnosis of mania plus SARS-CoV-2 infection. Seven patients had a preexisting diagnosis of BD (21, 29); two patients had a preexisting diagnosis of major depressive disorder/unipolar depression (21, 31) 1 patient had a preexisting diagnosis of OCD (33), 2 of psychosis (21). Some cases, despite having a negative psychiatric history, had a positive psychiatric family history: 3 for BD (27, 28, 35) 1 for cognitive deficits, major depressive disorder and delirium (31). Since the aim of the review was to identify episodes of mania in the context of Covid-19, single patient outcome (resolution of the disease, remission, etc.) were not taken into consideration. Different and common clinical aspects were included in the main table in the symptoms column. Not all articles mentioned lab results of the case presented. Whenever mentioned, the mostly involved inflammatory markers were fibrinogen, ferritin, LDH, d-dimer, PCR, ESR, IL-6, IL-10.

## Discussion

There is increasing evidence in literature regarding patients infected with SARS-CoV-2 which developed manic symptoms, possibly correlating with the infection itself, due to a cascade triggered by SARS-CoV-2 neuroinvasion, increased neuroinflammatory and inflammatory response in general, hypoxia, iatrogenic factors including antibiotics and steroidal therapy. Results of our research raise questions regarding the possibility that SARS-CoV-2 infection can be a trigger for a manic/hypomanic episode. On the basis that COVID-19 is still under many investigations regarding all consequences of the disease, the study can be enlightening in helping clinicians to suspect a psychiatric correlate of COVID-19 when specific symptoms of mania arise.

Iqbal et al. (21) have highlighted how mania can both be caused by psychosocial stress in susceptible individuals, and by an inflammatory mechanism as well, as has been hypothesized by Park et al. (25) as well: COVID-19 infection could trigger an initial manic episode, SARS-CoV-2 could penetrate blood–brain barriers and stimulate the production of cytokines (TNF-α, IL 1 and 6, and INF-α). Nonetheless, DSM-5 (38) and ICD-10 (39) permit a diagnosis of manic episode even in the background of a medical disorder or substance use, and this could be the case with COVID-19, but knowing how widespread the virus has become, this could influence the incidence of manic episodes across all countries.

The main mania symptoms experienced in the cases analyzed include classical presentations of mania: insomnia (22, 26) abnormal behavior (23, 24, 27), delusions (30, 31), irritability (34, 36), agitation (35), aggressive behavior (33, 37), anxiety (37), impaired concentration (21), persecutory beliefs (30), auditory hallucinations (21, 25), grandiose ideas (21, 28, 29). These findings support the hypothesis that the psychiatric condition experienced is no other than a manic episode, therefore not representing a separate diagnostic entity. The fact that remission of the mood swing occurred after appropriate medical treatment (mostly thanks to Atypical and Typical Anti-psychotics) further supports this statement.

The elevated peripheral inflammatory markers support neuroinflammation as a possible mechanism for COVID-19 causing the patients' neuropsychiatric symptoms (22, 26, 29). Furthermore, the included studies showed that the severity of COVID-19 did not correlate with manic symptoms, suggesting hidden neuroinflammatory mechanisms are strongly present. A discrepancy between pro-inflammatory and anti-inflammatory cytokines has been observed in bipolar patients, the former being more elevated and the latter being less expressed, in particular during mania. (40) Inflammation may act as a triggering factor by disrupting the blood brain barrier mostly, allowing SARS-CoV-2 entrance in the CNS (41), although the neurotropism of SARS-CoV-2 is still under investigation (42). SARS-CoV-2 apparently enters human cells through ACE-2, which is mostly expressed in the respiratory and gastrointestinal system, although being present in the endothelial system as well, also in the brain (10, 11). Invasion of the Central Nervous and of the Peripheral Nervous System as well (43) may explain a potential psychiatric clinical presentation of COVID-19. More specifically, in animal models there is evidence of the expression of ACE-2 in the amygdala, i.e., a site in which the spike proteins of the virus may bind (44). Neuronal and endothelial cells are potential targets for SARS-CoV-2 infection, which may cause their dysregulation after contact with the Spike viral protein (45). Viral invasion of the CNS may occur through several routes, including transsynaptic transfer across infected neurons, entry via the olfactory nerve, vascular endothelium infection, and leukocyte migration across the blood-brain barrier (46). Furthermore, SARS-CoV-2 can replicate in *in-vitro* neuronal cells, although confirmatory *in vivo* studies are required (47).

Worth of mention is that behavioral and mood disorders following infectious disorders have been observed previously as far as other coronaviruses are concerned (48). Studies have shown that individuals affected by BD may show an increased inflammatory status (49), therefore neuroinvasion may act as a trigger for the disease in those with a certain predisposition, culminating with the well-known manifestations of mania. Knowing the role played by the inflammatory cascade in the development of BD (50, 51), the hypothesis that COVID-19 may be a co-protagonist in unmasking a latent BD, as has happened in some cases analyzed (27, 28, 31, 35, 36) is gaining importance, yet needing further investigation.

Regarding the hypothesis that the pandemic social consequences themselves can be triggering factors for a previously unknown BD [as happened in some case reports analyzed: Uzun et al. (36), Varsak et al. (37), Meltem et al. (32)] or an exacerbating factor for those who had already been diagnosed with BD (52, 53), this appears to be relevant, as previous studies have already shown that social disasters can exacerbate BD mania symptoms but no other psychiatric disorders (54). Lockdown measures adopted to embank the pandemic influenced some of those factors which are crucial in mood maintenance in BD, such as sleep and social interactions, therefore possibly causing exacerbation of the mood disorder (55). In fact, studies have shown how the current pandemic has led to more depressive episodes in individuals affected by BD (56) compared to controls and patients affected by unipolar depression (57).

## Limits

Our investigation was based on a low sample population and evidence mainly comes from single case reports and case series. Hence, further investigation is needed as a direct correlation between mania and SARS-CoV-2 neuroinvasion cannot be stated with certainty, although having some clue.

## Conclusions

Manic episodes occurring in the context of COVID-19 are becoming more and more frequent. Knowing that biopsychological factors and environmental factors all concur in the development and/or exacerbation of BD, already well known to be of multifactorial etiology, raises alarm because of the high incidence of SARS-CoV-2 infections throughout the world. Awareness should be raised in physicians witnessing symptoms of mania in patients affected by COVID-19, even if asymptomatic from the organic point of view, and even if the patient has no prior psychiatric history. Results have shown that COVID-19 may trigger a pre-existing bipolar disorder or unmask an unknown BD, due to social and psychological influences and through biological pathways both. Further research is needed to understand the precise mechanisms of neurotropism of SARS-CoV-2 and, hopefully, prevent it at least in those patients who already received a diagnosis of BD. These, in particular, need a certain clinical focus, as they are more prone to re-exacerbation of the disease due to the stress which followed the pandemic, social isolation, difficulties in receiving appropriate medical attention and follow-up, therapy changes due to Covid-19 disease, increased inflammatory response and SARS-CoV-2 neuroinvasion of the CNS.

## Author Contributions

AD, RT, and MNM: conceptualization. AD and LR: data curation. AD, LR, MNM, and PG: investigation. AD: methodology. AD, PG, and RT: supervision. AD, MNM, and LR: roles/writing—original draft. AD and MNM: writing—review and editing. All authors contributed to the article and approved the submitted version.

## Conflict of Interest

The authors declare that the research was conducted in the absence of any commercial or financial relationships that could be construed as a potential conflict of interest.

## Publisher's Note

All claims expressed in this article are solely those of the authors and do not necessarily represent those of their affiliated organizations, or those of the publisher, the editors and the reviewers. Any product that may be evaluated in this article, or claim that may be made by its manufacturer, is not guaranteed or endorsed by the publisher.
